# Occurrence, molecular confirmation, and multidrug resistance of methicillin-resistant *Staphylococcus aureus* and *Staphylococcus pseudintermedius* in companion animals in Indonesia

**DOI:** 10.14202/vetworld.2026.324-338

**Published:** 2026-01-25

**Authors:** Ghias Ghifari Alhadz, Siti Isrina Oktavia Salasia, Fajar Budi Lestari, Alyaa Rifqoh Putri Yosyana, Madarina Wasissa, Yasinta Rahma Setianingrum, Rini Widayanti

**Affiliations:** 1 Department of Clinical Pathology, Faculty of Veterinary Medicine, Universitas Gadjah Mada, Yogyakarta, Indonesia; 2 Department of Bioresources Technology and Veterinary, Vocational College, Universitas Gadjah Mada, Yogyakarta, Indonesia; 3 Department of Biochemistry, Faculty of Veterinary Medicine, Universitas Gadjah Mada, Yogyakarta, Indonesia

**Keywords:** Antimicrobial resistance, companion animals, Indonesia, *mecA* gene, methicillin-resistant *Staphylococcus aureus*, methicillin-resistant *Staphylococcus pseudintermedius*, multidrug resistance, One Health

## Abstract

**Background and Aim:**

Methicillin-resistant *Staphylococcus aureus* (MRSA) and *Staphylococcus pseudintermedius* (MRSP) are increasingly recognized as important pathogens in companion animals, with significant zoonotic and public health implications. Data on methicillin-resistant staphylococci in pets in Indonesia remain scarce, particularly from clinical settings. This study aimed to determine the occurrence, molecular identity, and antimicrobial resistance (AMR) profiles of MRSA and MRSP isolated from companion animals presenting with clinical infections using an integrated phenotypic and genotypic diagnostic approach.

**Materials and Methods:**

We collected 100 clinical swab samples from dogs (n = 26), cats (n = 67), and rabbits (n = 7) presenting with signs of bacterial infection at veterinary clinics in Central Java and the Special Region of Yogyakarta, Indonesia. Isolates were identified using standard biochemical tests and confirmed molecularly by PCR targeting the 23S rRNA and *nuc* genes for *S. aureus* and PCR–restriction fragment length polymorphismof the *pta* gene for *S. pseudintermedius*. Methicillin resistance was screened using oxacillin resistance screening agar base, phenotypically confirmed by disk diffusion (cefoxitin or oxacillin), and genotypically verified by detection of the *mecA* gene. The Kirby–Bauer method was used to perform antimicrobial susceptibility testing against 11 commonly used antibiotics.

**Results:**

Of the 100 samples, 41 *S. aureus* and 14 *S. pseudintermedius* isolates were confirmed. Based on *mecA* detection, 27/41 (65.9%) *S. aureus* isolates were classified as MRSA and 13/14 (92.9%) *S. pseudintermedius* isolates were classified as MRSP. MDR was highly prevalent, observed in 92.6% of MRSA and 92.3% of MRSP isolates. High resistance rates were noted against β-lactam antibiotics, including penicillin, ampicillin, and amoxicillin. Several isolates carried mecA despite being phenotypically susceptible, indicating silent or low-expression resistance determinants.

**Conclusion:**

This study reveals a great burden of methicillin- and multidrug-resistant staphylococci among companion animals with clinical infections in Indonesia. The detection of *mecA*-positive MRSA and MRSP underscores a substantial zoonotic risk and highlights the limitations of phenotypic methods. These findings emphasize the need for routine molecular diagnostics, strengthened antimicrobial stewardship in veterinary practice, and integrated One Health surveillance to mitigate the spread of AMR across animal–human interfaces.

## INTRODUCTION

Staphylococci are opportunistic pathogens capable of infecting both humans and animals and are associated with a wide range of clinical conditions [1]. Among companion animals, *Staphylococcus aureus* is the most frequently encountered pathogenic species and represents a major infectious agent responsible for substantial morbidity and mortality worldwide [2, 3]. In animals, S. aureus has been implicated in mastitis, omphalitis, botryomycosis, and bacteremia [4]. Another clinically important species, *Staphylococcus pseudintermedius*, is also commonly isolated from animals [1]. Previous investigations have demonstrated that *S. pseudintermedius* can be recovered from dogs, cats, and horses, with reported prevalence rates ranging from 37% to 92% [5, 6]. In companion animals, this bacterium is frequently associated with pyoderma, otitis externa, ophthalmic infections, pyometra, and urinary tract infections [5, 7–9].

Methicillin-resistant *S. aureus* (MRSA) is regarded as one of the most significant bacterial pathogens because of its ability to withstand multiple antimicrobial classes. MRSA has been identified as the causative agent in approximately 25% of nosocomial infections, ranging from mild superficial skin infections to severe, life-threatening septicemia [10]. Kasela *et al*. [11] reported MRSA detection rates of 39.3% in dogs and 26.5% in cats. Similarly, methicillin-resistant *S. pseudintermedius* (MRSP) was first documented in North America in 2001 and later reported in Europe in 2007 [12].

Methicillin resistance in both MRSA and MRSP is primarily mediated by the *mecA* gene, which encodes penicillin-binding protein 2a (PBP2a) [6, 13]. This altered protein exhibits a markedly reduced affinity for β-lactam antibiotics, thereby rendering these agents ineffective. The multidrug-resistant (MDR) nature of MRSA and MRSP has contributed to outbreaks that are increasingly difficult to control. MRSA is now classified as an emerging infectious pathogen capable of spreading through direct contact between colonized or infected healthcare personnel and patients, thereby contributing to nosocomial infections [14]. Comparable nosocomial transmission events have also been reported in veterinary settings, raising serious concerns for veterinary hospitals, particularly in the United States [10].

Human infections caused by MRSP have also been documented and are often linked to close contact with companion animals, especially dogs. However, the true prevalence of MRSP colonization in humans remains unclear because this organism is frequently misidentified as *S. aureus* [15]. Up to 2023, a total of 87 human infections caused by *S. pseudintermedius* have been reported across 32 countries. Contact studies have shown that MRSP was detected in 36% (5/14) of dogs and 31% (4/13) of cats, whereas 3% (4/141) of veterinary personnel tested positive. In a veterinary hospital in Sydney, Australia, 4% of staff were reported to carry MRSA, while 7% of canine patients carried MRSP [15]. The close and frequent interaction between humans and companion animals through daily contact creates a potential risk for interspecies transmission of MRSA and MRSP [13]. The presence of these resistant strains is of major concern because infections caused by such bacteria may delay wound healing and increase both morbidity and mortality [16].

Despite growing global concern, reports on MRSA and MRSP in companion animals in Indonesia remain limited. Most Indonesian studies have focused on MRSA in livestock, food-chain samples, or human clinical isolates, whereas data from pets are scarce. To date, surveillance data derived from companion animals attending Indonesian veterinary hospitals are virtually absent, and no published studies have documented the local prevalence or molecular characteristics of MRSA or MRSP in dogs, cats, or rabbits. Moreover, accurate differentiation of *S. pseudintermedius* from other members of the *Staphylococcus intermedius* group using Polymerase chain reaction–restriction fragment length polymorphism (PCR–RFLP) analysis of the *pta* gene with *Mbo*I digestion is rarely applied in Indonesian veterinary diagnostics. The application of this method, which yields the characteristic dual bands of 213 and 107 bp, therefore represents an important technical novelty.

Additionally, the detection of *mecA*-positive but phenotypically susceptible isolates constitutes an important and underreported phenomenon in Indonesia, suggesting the presence of silent or low-expression *mecA* elements in pet-associated staphylococci. Such silent or low-expression variants may lead to underdiagnosis of MRSA or MRSP when laboratories rely exclusively on phenotypic methods, thereby posing potential risks for clinical decision-making in veterinary practice. Given these diagnostic challenges and the paucity of local clinical data, a substantial knowledge gap exists regarding the occurrence, molecular identity, and antimicrobial resistance (AMR) patterns of MRSA and MRSP in companion animals in Indonesia. From a One Health perspective, this gap also limits the ability to detect and mitigate potential cross-species transmission of methicillin-resistant staphylococci among pets, owners, and veterinary personnel. Because companion animals share household environments and clinical spaces with humans, undetected MRSA or MRSP may contribute to the silent circulation of antimicrobial-resistant pathogens within the community.

Therefore, the present study was designed to investigate the occurrence, molecular identity, and AMR characteristics of MRSA and MRSP isolated from companion animals presenting with clinical infections in Indonesia. Specifically, this study aimed to (i) isolate and phenotypically identify *S. aureus* and *S. pseudintermedius* from dogs, cats, and rabbits attending veterinary clinics; (ii) confirm species identity using molecular methods, including PCR targeting the *23S rRNA* and *nuc* genes for *S. aureus* and PCR–RFLP analysis of the *pta* gene for accurate differentiation of *S. pseudintermedius* from other members of the *Staphylococcus intermedius* group; (iii) determine methicillin resistance through an integrated diagnostic approach combining oxacillin resistance screening agar base, disk diffusion testing, and detection of the *mecA* gene; and (iv) characterize the antimicrobial susceptibility and multidrug resistance (MDR) profiles of the confirmed isolates. By generating baseline molecular and phenotypic data from companion animals, this study seeks to address critical knowledge gaps in Indonesian veterinary medicine and to contribute evidence relevant to antimicrobial stewardship, infection control, and One Health–based surveillance of methicillin-resistant staphylococci at the animal–human interface.

## MATERIALS AND METHODS

### Ethical approval

This study was approved by the Ethics Committee of the Faculty of Veterinary Medicine, Universitas Gadjah Mada, Indonesia (Approval No. EC 143/EC-FKH/Int./2024). Written informed consent was obtained from all animal owners prior to sample collection using standard clinic consent forms. Clinical samples were collected as part of routine veterinary diagnosis, and no additional experimental procedures were performed.

Sample collection involved non-invasive swabbing (nasal, skin/wound, ear, ocular, and perineal) conducted by trained veterinarians using aseptic techniques, causing minimal discomfort and requiring no sedation or euthanasia. Animal welfare was prioritized at all stages. All laboratory work was carried out in accordance with biosafety guidelines for handling potentially zoonotic pathogens. Animal and owner data were anonymized and used solely for the purposes outlined in the approved study protocol.

### Study design, period, and location

This cross-sectional study was conducted between September 2024 and July 2025 at the Clinical Pathology Laboratory, Faculty of Veterinary Medicine, and the Animal Science Learning Center (ASLC), Universitas Gadjah Mada, Yogyakarta, Indonesia. A total of 100 clinical swab samples were collected from 26 dogs, 67 cats, and 7 rabbits presenting to veterinary clinics with clinical signs consistent with bacterial infection. Samples were obtained from 11 veterinary clinics and hospitals located in Central Java and the Special Region of Yogyakarta, Indonesia. All animals enrolled in the study exhibited clinical manifestations suggestive of bacterial infection.

### Inclusion and exclusion criteria

Animals presenting with skin or soft-tissue lesions, otitis externa, nasal discharge, conjunctivitis, perineal infection, or wound-related inflammation were eligible for inclusion. Animals that had received systemic antibiotic treatment within the preceding 7 days or had incomplete clinical records were excluded. Duplicate samples collected from the same anatomical site of an individual animal were not included in the analysis.

### Clinical data collection and sample handling

**Figure 1:**
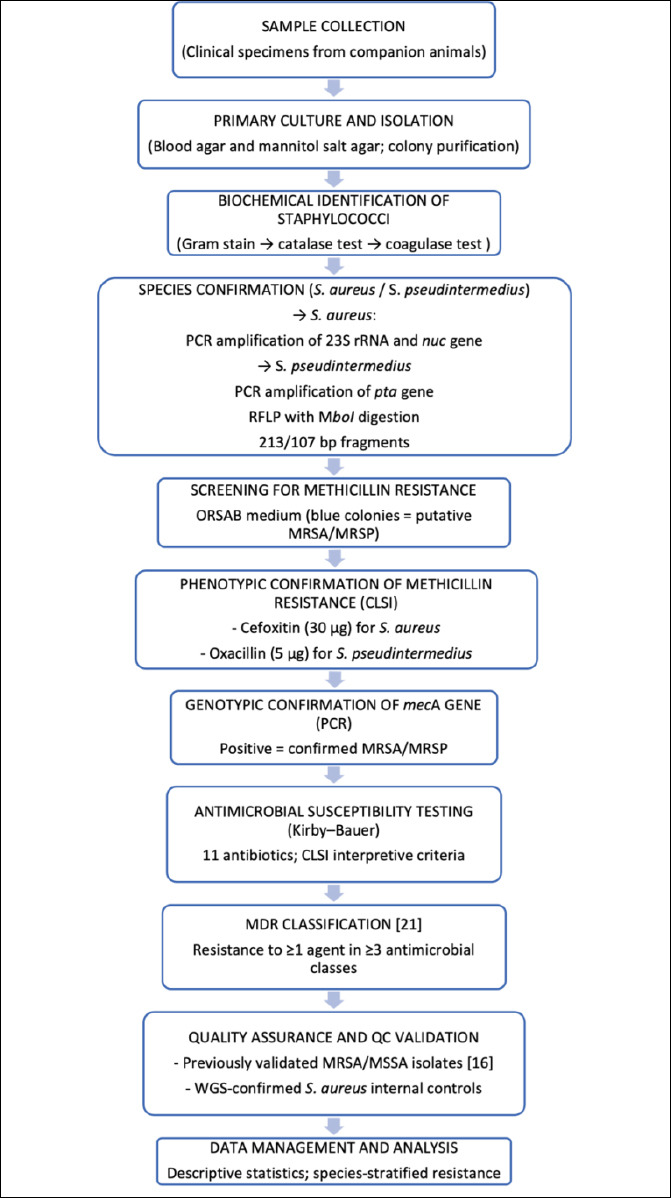
Workflow diagram illustrating the integrated phenotypic and molecular approach used for isolation, identification, and antimicrobial resistance characterization of methicillin-resistant *Staphylococcus aureus* and *Staphylococcus pseudintermedius* from companion animals.

For each animal, information on species, age, sex, and, when available, breed was recorded along with the clinical diagnostic category. A targeted symptomatic sampling strategy was applied to ensure the inclusion of clinically relevant *Staphylococcus* isolates. Clinical specimens, including skin, nasal, ear, ocular, and perineal swabs, were aseptically collected using sterile rayon swabs and placed in Amies transport medium. Samples were transported under refrigerated conditions (4°C–8°C) and processed within 4–6 h of collection. When samples were collected from multiple anatomical sites of the same animal, only the site most representative of the lesion was selected to ensure that each isolate originated from a unique animal. The overall study workflow is illustrated in Figure 1.

### Phenotypic identification of *Staphylococcus* spp.

All clinical swab samples were inoculated onto Mannitol Salt Agar (MSA) (MerckMillipore™, Germany) and incubated at 37°C for 24 h. Colony morphology was examined, followed by Gram staining and catalase testing. Pure colonies were subsequently subjected to coagulase, ortho-nitrophenyl-β-galactoside (ONPG) (Himedia™, India), and Voges–Proskauer (VP) (Himedia™) tests to differentiate *S. aureus* from *S. pseudintermedius* after incubation at 37°C for 24–48 h. VP-positive isolates produced a red coloration at the top of the medium, whereas VP-negative isolates showed no color change. *S. aureus* is VP-positive, while *S. pseudintermedius* is VP-negative. In the ONPG test, positive isolates produced a yellow coloration, whereas negative isolates remained clear; *S. pseudintermedius* is ONPG-positive, whereas *S. aureus* is ONPG-negative [1, 16]. Hemolytic activity was evaluated using 5% sheep blood agar plates (4 mm thickness) (MerckMillipore™) following incubation at 37°C for 24 h.

### Genomic DNA extraction

Genomic DNA was extracted from confirmed *S. aureus* and *S. pseudintermedius* isolates using the DNA Isolation Kit (Geneaid Biotech Ltd., Taiwan) according to the manufacturer’s instructions. Extracted DNA was stored at −20°C until further molecular analysis.

### PCR amplification for species confirmation

Polymerase chain reaction (PCR) assays targeting the 23S rRNA and nuc genes were performed to confirm *S. aureus* isolates. The 23S rRNA gene is a conserved component of the bacterial ribosome and is commonly used for genus- and species-level identification [16]. The *nuc* gene encodes a thermonuclease virulence factor and is widely used as a molecular marker to distinguish S. aureus from other *Staphylococcus* species. Identification of *S. pseudintermedius* was performed by targeting the *pta* gene, which contains a restriction site for the *Mbo*I enzyme that is absent in other members of the *S. intermedius* group (SIG). Therefore, *pta* gene detection was followed by PCR–RFLP analysis to differentiate *S. pseudintermedius* from other SIG members.

Each 25 μL PCR reaction mixture consisted of 1 μL of forward primer, 1 μL of reverse primer, 12.5 μL of MyTaq Red Mix, 2 μL of DNA template, and 8.5 μL of nuclease-free water. Amplification was carried out using a thermal cycler (Benchmark Scientific, Inc.). PCR products were resolved by electrophoresis on 1.5% agarose gels stained with RedSafe (Intron Biotechnology, Korea) at 100 V for 30 min using Tris–borate–ethylenediamine-tetraacetic acid buffer and visualized under a UV transilluminator [16–18]. Primer sequences and amplification conditions are summarized in Table 1 [16, 18].

**Table 1:** Oligonucleotide primers and polymerase chain reaction conditions used in this study.

Target gene	Primer sequence (5’-3’)	Amplicon size (bp)
23S rRNA (*Staphylococcus aureus*)	AGC GAG TTA CAA AGG ACG AC	1250^1^
	AGC TCA GCC TTA ACG AGT AC	
*nuc* (*S. aureus*)	GCG ATT GAT GGT GAT ACG GTT ACG CAA GCC TTG ACG AAC TAA AGC	267^2^
*pta* (*Staphylococcus pseudintermedius*)	AAA GAC AAA CTT TCA GGT AA GCA TAA ACA AGC ATT GTA CCG	320^3^
*mecA* (*MRSA/MRSP*)	AAAATCGATGGTAAAGGTTGGC AGTTCTGCAGTACCGGATTTGC	532^4^

1 35 cycles of 95°C for 15 s, 64°C for 30 s, and 72°C for 10 s [16].

2 37 cycles of 94°C for 60 s, 55°C for 30 s, and 72°C for 5 s [16].

3 30 cycles of 95°C for 60 s, 53°C for 30 s, and 72°C for 30 s [18].

4 35 cycles of 95°C for 30 s, 55°C for 30 s, and 72°C for 10 s [16].

### PCR–RFLP analysis of the *pta* gene for *S. pseudintermedius* identification

Amplified *pta* gene products were subjected to RFLP analysis using the *Mbo*I restriction enzyme (10 U/µL; Thermo Fisher Scientific, Waltham, MA, USA). For digestion, 25 µL of PCR product was mixed with 0.5 µL (5 U) of *Mbo*I and 5 µL of 5× digestion buffer and incubated at 37°C for 2 h, corresponding to the optimal activity temperature of the enzyme. Digested products were separated on 1.5% agarose gels (GeneDirex, USA) using a 50 bp DNA ladder (Invitrogen, USA) and visualized under UV illumination.

The 320 bp *pta* gene fragment of *S. pseudintermedius* contains an *Mbo*I restriction site, resulting in two fragments of 213 bp and 107 bp following digestion, whereas other SIG members lack this site and remain undigested [18, 19].

### Antimicrobial susceptibility testing (Kirby–Bauer method)

Antimicrobial susceptibility testing was performed using the Kirby–Bauer disk diffusion method in accordance with CLSI M100 guidelines [19]. The antimicrobial agents tested included oxacillin (5 µg), tetracycline (30 µg), erythromycin (15 µg), cefoxitin (30 µg), ciprofloxacin (5 µg), gentamicin (10 µg), ampicillin (10 µg), clindamycin (10 µg), penicillin G (10 µg), vancomycin (30 µg), and amoxicillin (25 µg). Mueller–Hinton agar plates were maintained at a uniform thickness of 4 mm. Bacterial suspensions were vortexed for 15–20 s, adjusted to 0.5 McFarland turbidity, and evenly inoculated onto agar surfaces using sterile cotton swabs. Plates were incubated at 37°C for 24 h, after which inhibition zone diameters were measured in millimeters and interpreted as susceptible, intermediate, or resistant following CLSI M100 criteria [19].

### Screening and confirmation of methicillin resistance

Isolates identified as *S. aureus* or *S. pseudintermedius* were initially screened for methicillin resistance using Oxacillin Resistance Screening Agar Base (ORSAB; Oxoid, UK), a chromogenic medium containing oxacillin and mannitol indicators. The formation of distinct blue colonies was interpreted as presumptive MRSA or MRSP. ORSAB contains oxacillin (2 mg/L) and polymyxin B (50,000 IU/L), allowing selective growth of methicillin-resistant isolates. Blue colony coloration indicates mannitol fermentation and uptake of the aniline dye present in the medium.

Phenotypic confirmation of methicillin resistance was performed by disk diffusion testing using cefoxitin (30 µg) for *S. aureus* and oxacillin (5 µg) for *S. pseudintermedius* in accordance with CLSI M100 guidelines [19]. Genotypic confirmation was achieved by PCR amplification of the mecA gene using specific primers (Table 1). Isolates positive for *mecA* were classified as MRSA or MRSP [20]. MDR was defined as resistance to at least one antimicrobial agent in three or more antimicrobial classes, following the criteria of Magiorakos *et al*. [21].

### Quality control strains

In addition to standard reference strains, previously characterized *S. aureus* isolates reported by Fitranda *et al*. [16], including validated MRSA and MSSA strains, were used as quality control (QC) isolates. These strains had been confirmed by biochemical identification, cefoxitin or oxacillin susceptibility testing, and *mecA* PCR and were routinely employed to verify culture performance, PCR accuracy, and antimicrobial susceptibility testing reproducibility. The QC *S. aureus* isolates had undergone whole-genome sequencing (WGS) as part of laboratory validation, providing high-resolution confirmation of species identity and resistance gene content, thereby ensuring methodological robustness and minimizing analytical variation.

### Data management and statistical analysis

All data were compiled and cleaned using Microsoft Excel 2021 and analyzed using IBM SPSS Statistics version 26 (IBM Corp., Armonk, NY, USA) and GraphPad Prism version 10 (GraphPad Software, San Diego, CA, USA).. Continuous variables are presented as mean ± standard deviation, while categorical variables, including species distribution, prevalence of *S. aureus* and *S. pseudintermedius*, and proportions of MRSA, MRSP, and MDR isolates, are expressed as frequencies and percentages. Percentages were calculated using both overall sample denominators (n = 100) and species-specific denominators for AMR analyses. Ninety-five percent confidence intervals (95% CI) were calculated for key prevalence estimates. Comparative analyses between species and sample types were conducted using the chi-square test or Fisher’s exact test when expected cell counts were <5. Statistical significance was set at p < 0.05.

## RESULTS

### Phenotypic identification and sample characteristics

A total of 100 clinical swab samples were collected from 67 cats (67%), 26 dogs (26%), and 7 rabbits (7%). Most animals presented with mild to moderate clinical infections, including skin or soft-tissue lesions, otitis externa, respiratory or nasal discharge, conjunctivitis, and perineal dermatitis. The median age of the sampled animals was 1.5 years (range: 1 month–14 years). Overall, 58% of the animals were male and 42% were female. Sample types consisted of nasal swabs (42%), skin swabs (19%), ear swabs (18%), ocular swabs (16%), and perineal swabs (5%). A summary of sample categories and demographic characteristics is presented in Table 2.

**Table 2:** *Staphylococcus* isolates obtained from companion animals, showing sample origin, demographic characteristics, molecular identification results, and antimicrobial resistance profiles.

No.	Sample code	Species/Age/Sex	Specimen	Sample source	*23S rRNA*	*nuc*	*pta*-RFLP	*mecA*	ORSAB	Hem	MDR type
1	S-K-12	Cat/6 mo/F	Nasal	ACS	+	+	–	+	+	β	MRSA-MDR
2	S-K-15	Cat/6 y/M	Nasal	ACS	+	+	–	+	+	β	MRSA-MDR
3	S-K-18	Cat/1 y/F	Ear	ACS	+	+	–	–	+	α	MRSA-MDR
4	S-K-19	Cat/1.5 y/M	Ear	ACS	–	–	+	+	+	β	MRSP-MDR
5	S-K-25	Cat/1 y 5 mo/F	Ear	ACS	+	+	–	–	+	β	MSSA-MDR
6	S-K-34	Cat/1 y/F	Ear	ACS	–	–	+	+	–	α	MRSP-MDR
7	S-K-35	Cat/3 y 7 mo/M	Ear	ACS	–	–	+	+	+	β	MRSP-MDR
8	S-K-37	Cat/2 y/F	Nasal	ACS	–	–	+	+	+	β	MRSP-MDR
9	K-1	Cat/1.5 y/M	Nasal	SAH	+	+	–	–	+	γ	MSSA-MDR
10	K-2	Cat/2 y/F	Nasal	SAH	+	+	–	–	+	γ	MSSA-MDR
11	K-22	Cat/–/M	Nasal	DC	+	+	–	+	+	γ	MRSA-MDR
12	K-25	Cat/2 y/M	Skin	KHJ	+	+	–	+	+	β	MRSA-MDR
13	K-26	Cat/1 y/F	Skin	KHJ	+	+	–	+	+	β	MRSA-MDR
14	K-29	Cat/2 y/M	Nasal	PPC	+	+	–	+	+	γ	MRSA
15	K-30	Cat/3 mo/F	Skin	PPC	+	+	–	+	–	β	MRSA-MDR
16	K-32	Cat/6 mo/F	Nasal	PPC	+	+	–	+	+	β	MRSA-MDR
17	K-34	Cat/3 mo/F	Nasal	PPC	+	+	–	+	+	γ	MRSA-MDR
18	K-35	Cat/–/–	Nasal	PPC	+	+	–	+	+	γ	MRSA-MDR
19	K-36	Cat/7 y/M	Nasal	SAH	+	+	–	–	+	β	MSSA-MDR
20	K-40	Cat/–/F	Skin	SAH	+	+	–	+	+	γ	MRSA-MDR
21	K-42	Cat/3 y/M	Nasal	PPC	+	+	–	+	+	β	MRSA-MDR
22	K-48	Cat/1.5 y/M	Eyes	PV	–	–	+	+	+	α	MRSP-MDR
23	K-51	Cat/–/–	Eyes	PV	+	+	–	+	+	β	MRSA-MDR
24	K-53	Cat/4 y/M	Nasal	PPC	+	+	–	–	+	γ	MSSA-MDR
25	K-55	Cat/3 y/M	Nasal	PPC	–	–	+	+	+	α	MRSP
26	K-57	Cat/1 y/F	Nasal	PPC	+	+	–	–	+	β	MSSA-MDR
27	K-59	Cat/1 y/F	Nasal	PPC	–	–	+	+	+	α	MRSP-MDR
28	K-62	Cat/1 y/M	Nasal	PPC	–	–	+	+	+	γ	MRSP-MDR
29	K-66	Cat/3 y/M	Nasal	APC	+	+	–	+	+	β	MRSA-MDR
30	K-71	Cat/3 y/M	Nasal	SK	–	–	+	+	–	β	MRSP-MDR
31	K-92	Cat/2 y/M	Nasal	PPC	+	+	–	–	+	β	MSSA-MDR
32	K-93	Cat/6 y/M	Nasal	PPC	+	+	–	–	+	β	MSSA-MDR
33	K-98	Cat/–/–	Skin	KK	+	+	–	–	+	β	MSSA-MDR
34	K-107	Cat/3 y/F	Nasal	SAL	+	+	–	+	+	α	MRSA-MDR
35	A-2	Dog/4 y/F	Perineum	SAL	–	–	+	+	+	α	MRSP-MDR
36	A-3	Dog/2 y/F	Perineum	SAL	–	–	+	+	+	α	MRSP-MDR
37	A-4	Dog/2 y/F	Perineum	SAL	–	–	+	–	+	α	MSSP-MDR
38	A-5	Dog/2 y/F	Skin	SAL	–	–	+	+	+	γ	MRSP-MDR
39	A-6	Dog/9 mo/F	Skin	SAL	+	+	–	+	+	γ	MRSA-MDR
40	A-7	Dog/–/M	Ear	SRRDC	+	+	–	+	+	β	MRSA-MDR
41	A-8	Dog/–/F	Ear	SRRDC	–	–	–	+	+	β	MRSP-MDR
42	A-10	Dog/–/F	Perineum	SRRDC	+	+	–	+	+	γ	MRSA
43	A-15	Dog/6 y/M	Skin	SRRDC	+	+	–	–	+	β	MSSA-MDR
44	A-17	Dog/9 mo/F	Skin	PPC	+	+	–	+	+	γ	MRSA-MDR
45	A-18	Dog/6 mo/M	Eyes	SAH	+	+	–	–	+	γ	MSSA-MDR
46	A-25	Dog/3 y/F	Eyes	SAH	+	+	–	–	+	γ	MSSA-MDR
47	A-27	Dog/14 y/M	Eyes	SAH	+	+	–	+	+	β	MRSA-MDR
48	A-28	Dog/–/–	Eyes	SAH	+	+	–	+	+	β	MRSA-MDR
49	A-29	Dog/3 mo/F	Skin	PPC	+	+	–	+	+	β	MRSA-MDR
50	R-1	Rabbit/4 mo/M	Skin	CP	+	+	–	+	+	β	MRSA-MDR
51	R-3	Rabbit/3.5 mo/F	Skin	CP	+	+	–	–	+	β	MSSA-MDR
52	R-4	Rabbit/10 mo/M	Skin	CP	+	+	–	–	+	β	MSSA
53	R-5	Rabbit/1 y/F	Skin	CP	+	+	–	+	+	γ	MRSA-MDR
54	R-6	Rabbit/1 y/F	Skin	CP	+	+	–	+	+	β	MRSA-MDR
55	R-7	Rabbit/7 mo/F	Skin	CP	+	+	–	+	+	γ	MRSA-MDR

ACS = Animal Center Semarang, APC = Arisna Pet Care, Cat = 34 isolates, CP = Clinical Pathology, DC = DJIO Clinic, Dog = 15 isolates, F = female, Hem = hemolysis, KHJ = Klinik Hewan Jogja, KK = Kitkat, M = male, MDR = multidrug-resistant isolate (resistant to ≥3 antimicrobial classes), mo = month, MRSA = Methicillin-resistant *Staphylococcus aureus*, MRSP = Methicillin-resistant *Staphylococcus pseudintermedius*, MSSA = Methicillin-susceptible *S. aureus*, MSSP = Methicillin-susceptible *S. pseudintermedius*, ORSAB = Oxacillin resistance screening agar base, PPC = Panda Pet and Care, PV = Provet, Rabbit = 6 isolates, SAH = Soeparwi Animal Hospital, SAL = Shelter Animalover, SK = Satwakita, SRRDC = Shelter RRDC, y = year (total isolates = 55).

### Species identification and molecular confirmation

**Figure 2:**
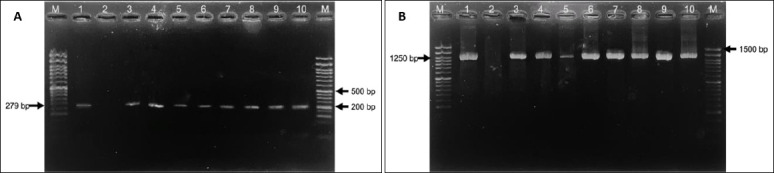
**(A**) Agarose gel electrophoresis of polymerase chain reaction products showing amplification of the *23S rRNA* gene (1250 bp). Lane M: 50 bp DNA ladder; lane 1: positive control; lane 2: negative control; lanes 3–10: representative *Staphylococcus aureus* isolates with specific target amplification. (B) Agarose gel electrophoresis of polymerase chain reaction products showing amplification of *nuc* gene (279 bp). Lane M: 50 bp DNA ladder; lane 1: positive control; lane 2: negative control; lanes 3–10: representative *Staphylococcus aureus* isolates with specific target amplification

**Figure 3:**
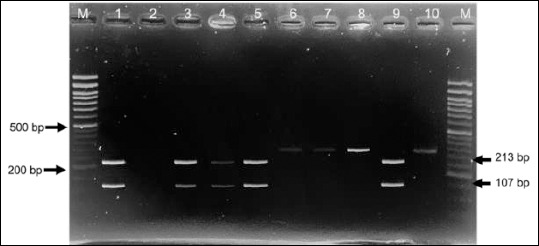
Polymerase chain reaction–restriction fragment length polymorphism analysis of the *pta* gene showing the characteristic 213- and 107-bp fragments of *Staphylococcus pseudintermedius*. Lane M: 50-bp DNA ladder; lane 1: positive control; lane 2: negative control; lanes 3–10: representative isolates displaying the expected digestion pattern.

Based on biochemical characteristics, 41 isolates (41%) were identified as *S. aureus* and 14 isolates (14%) as *S. pseudintermedius*. Molecular confirmation was subsequently performed. *S. aureus* isolates were verified by PCR amplification of the *23S rRNA* (1250 bp) and *nuc* (279 bp) genes (Figure 2). In contrast, *S. pseudintermedius* isolates were confirmed using PCR–RFLP analysis of the *pta* gene with the *Mbo*I restriction enzyme, which yielded two characteristic fragments of 213 bp and 107 bp (Figure 3).

### Distribution of isolates by sample origin

**Figure 4:**
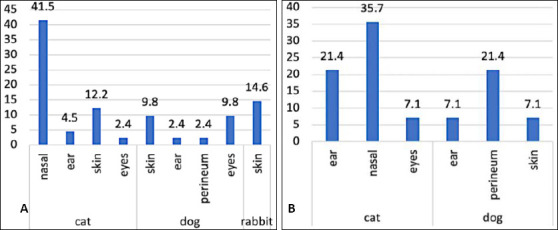
Distribution of confirmed (A) *Staphylococcus aureus* and (B) *Staphylococcus pseudintermedius* isolates by sample origin. The figure presents the number and proportion of isolates recovered from nasal, skin, wound, ear, ocular, and perineal specimens, highlighting the predominance of *S. aureus* in nasal swabs and *S. pseudintermedius* in skin and wound samples.

Figure 4 illustrates the distribution of confirmed *S. aureus* and *S. pseudintermedius* isolates according to sample origin. *S. aureus* was most frequently isolated from feline nasal swabs (17/41; 41.46%), followed by rabbit skin swabs (6/41; 14.63%), feline skin swabs (5/41; 12.20%), canine skin swabs (4/41; 9.76%), canine ocular swabs (4/41; 9.76%), feline ear swabs (2/41; 4.88%), canine ear swabs (1/41; 2.44%), and feline ocular swabs (1/41; 2.44%).

### Hemolytic activity of *Staphylococcus* isolates

Assessment of hemolytic activity on sheep blood agar revealed heterogeneous patterns among isolates. *S. aureus* isolates exhibited predominantly β-hemolysis in 56.10% (23/41), followed by γ-hemolysis in 39.02% (16/41) and α-hemolysis in 4.88% (2/41). In contrast, *S. pseudintermedius* isolates most frequently demonstrated α-hemolysis (57.14%, 8/14), followed by β-hemolysis (28.57%, 4/14) and γ-hemolysis (14.29%, 2/14).

These findings indicate that nasal swabs from cats and skin samples from rabbits were the most common sources of *Staphylococcus* isolates. The observed differences in hemolytic patterns between *S. aureus* and *S. pseudintermedius* suggest species-specific expression of hemolysins that may be influenced by host adaptation. Further genomic characterization of hemolysin-associated genes was not conducted and is acknowledged as a limitation of this study.

### Antimicrobial susceptibility profiles of *Staphylococcus* isolates

**Figure 5:**
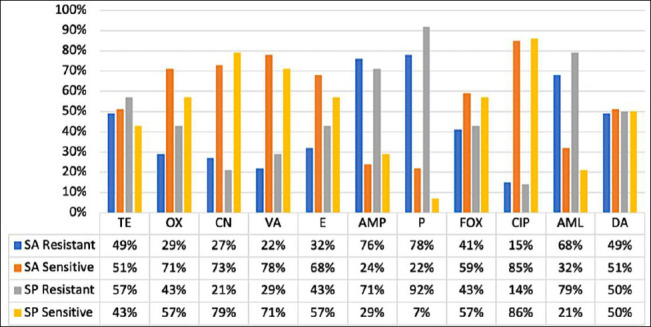
Antimicrobial susceptibility profiles of *Staphylococcus aureus* (SA) and *Staphylococcus pseudintermedius* (SP). The figure illustrates the proportions of SA and SP isolates classified as susceptible, intermediate, or resistant to the tested antibiotics, showing the highest resistance to β-lactams (penicillin, ampicillin, and oxacillin), whereas gentamicin and ciprofloxacin retained moderate to high activity. TE = Tetracycline, OX = Oxacillin, CN = Gentamicin, VA = Vancomycin, E = Erythromycin, AMP = Ampicillin, P = Penicillin, FOX = Cefoxitin, CIP = Ciprofloxacin, AML = Amoxicillin, DA = Clindamycin.

The antimicrobial susceptibility profiles of *S. aureus* and *S. pseudintermedius* isolates are summarized in Table 3 [19] and illustrated in Figure 5. Antimicrobial susceptibility testing was performed using the Kirby–Bauer disk diffusion method against 11 antibiotics representing multiple antimicrobial classes.

**Table 3:** Antimicrobial resistance and susceptibility profiles (mean ± SD) of *Staphylococcus* species.

=Species	Category	TE	OX	CN	VA	E	AMP	P	FOX	CIP	AML	DA
*Staphylococcus aureus*	R	1.11 ± 0.43	0.84 ± 0.50	0.23 ± 0.40	0.37 ± 0.26	0.33 ± 0.55	1.26 ± 0.77	1.08 ± 0.77	1.27 ± 0.75	0.27 ± 0.49	1.73 ± 0.47	0.46 ± 0.56
	S	3.08 ± 0.66	2.60 ± 0.74	1.74 ± 0.54	1.67 ± 0.18	2.38 ± 0.39	3.67 ± 0.54	3.79 ± 0.62	3.05 ± 0.38	2.50 ± 0.46	3.35 ± 0.58	2.68 ± 0.57
*Staphylococcus pseudintermedius*	R	1.10 ± 0.47	1.37 ± 0.15	0.30 ± 0.52	0.13 ± 0.23	0.00 ± 0.00	1.55 ± 0.95	1.79 ± 1.11	1.74 ± 0.55	0.00 ± 0.00	1.47 ± 1.30	0.19 ± 0.41
	S	2.61 ± 0.99	2.28 ± 1.16	2.10 ± 0.35	1.79 ± 0.14	2.13 ± 0.47	3.78 ± 0.71	3.50 ± 0.00	3.13 ± 0.50	3.04 ± 0.36	3.40 ± 0.53	2.64 ± 0.65

Values are expressed as mean ± SD. AML = Amoxicillin, AMP = Ampicillin, CIP = Ciprofloxacin, CN = Gentamicin, DA = Clindamycin, E = Erythromycin, FOX = Cefoxitin, OX = Oxacillin, P = Penicillin, R = Resistant, S = Susceptible, TE = Tetracycline, VA = Vancomycin. Reference: CLSI M100 (2023) guidelines [17].

Among *S. aureus* isolates, high resistance rates were observed against penicillin (78%), ampicillin (76%), and amoxicillin (68%). Moderate resistance was detected to clindamycin (49%), tetracycline (49%), cefoxitin (41%), erythromycin (32%), oxacillin (29%), gentamicin (27%), vancomycin (22%), and ciprofloxacin (15%).

Similarly, *S. pseudintermedius* isolates demonstrated pronounced resistance to penicillin (93%), amoxicillin (79%), and ampicillin (71%). Moderate resistance was observed to tetracycline (57%), clindamycin (50%), oxacillin (43%), cefoxitin (43%), erythromycin (43%), vancomycin (29%), gentamicin (21%), and ciprofloxacin (14%).

Overall, 93% of *S. aureus* (38/41) and *S. pseudintermedius* (13/14) isolates were classified as multidrug-resistant (MDR), exhibiting resistance to three or more antimicrobial classes. MDR was more prevalent among MRSA and MRSP isolates than among MSSA and MSSP isolates when stratified (Table 2). These findings highlight widespread β-lactam resistance and underscore the importance of prudent antimicrobial use in companion animals to limit the dissemination of resistant *Staphylococcus* strains. Minimum inhibitory concentration (MIC) testing was not performed and is acknowledged as a limitation.

### Screening and confirmation of methicillin resistance

Initial screening using Oxacillin Resistance Screening Agar Base (ORSAB) indicated a high proportion of presumptive methicillin-resistant isolates, with 95% (39/41) of S. aureus and 79% (11/14) of *S. pseudintermedius* producing characteristic blue colonies. However, phenotypic confirmation by disk diffusion yielded lower resistance rates, with only 41% (17/41) of *S. aureus* classified as MRSA based on cefoxitin resistance and 43% (6/14) of *S. pseudintermedius* classified as MRSP based on oxacillin resistance (Figure 5).

**Figure 6:**
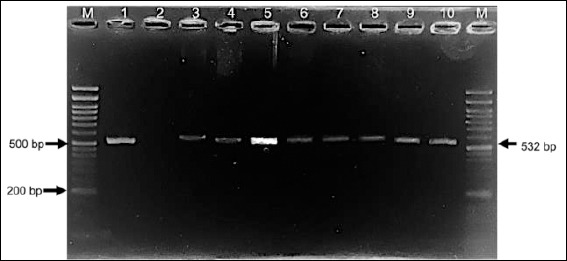
Agarose gel electrophoresis showing amplification of the *mecA* gene (532 bp) from selected *Staphylococcus aureus* and *Staphylococcus pseudintermedius* isolates. Lane M: 50-bp DNA ladder; lane 1: *S. aureus* *mecA*-positive reference strain; lane 2: negative control; lanes 3–10: representative *S. aureus* isolates.

Genotypic analysis provided greater sensitivity, with PCR detection of the *mecA* gene confirming methicillin resistance in 66% (27/41) of *S. aureus* and 93% (13/14) of *S. pseudintermedius* isolates through amplification of the 532 bp *mecA* fragment (Figure 6).

### Phenotype–genotype discordance in methicillin resistance

Comparison of screening, phenotypic, and genotypic methods revealed several phenotype–genotype discrepancies, including *mecA*-positive isolates that remained susceptible to cefoxitin or oxacillin and isolates exhibiting phenotypic resistance without detectable *mecA* (Table 4). These discordant profiles likely reflect heterogeneous expression of penicillin-binding protein 2a (PBP2a), borderline resistance, or alternative β-lactam resistance mechanisms.

**Table 4:** Methicillin resistance classification of *Staphylococcus aureus* and *Staphylococcus pseudintermedius* using three diagnostic methods (ORSAB screening, disk diffusion, and *mecA* polymerase chain reaction).

Method	*S. aureus* (n = 41)		*S. pseudintermedius* (n = 14)	
	
	MRSA	MSSA	MRSP	MSSP
ORSAB screening	39	2	11	3
Disk diffusion (FOX/OX)	17	24	6	8
*mecA* PCR	27	14	13	1

MRSA = Methicillin-resistant *Staphylococcus aureus*, MSSA = Methicillin-susceptible *Staphylococcus aureus*, MRSP = Methicillin-resistant *Staphylococcus pseudintermedius*, MSSP = Methicillin-susceptible *Staphylococcus pseudintermedius*, ORSAB = Oxacillin resistance screening agar base, FOX = Cefoxitin, OX = Oxacillin.

Notably, eleven *S. aureus* and seven *S. pseudintermedius* isolates were *mecA*-positive but phenotypically susceptible to cefoxitin or oxacillin. Such discrepancies have been previously reported in staphylococci isolated from companion animals and may result in underestimation of methicillin resistance when reliance is placed solely on phenotypic assays. In contrast, no isolates in this study were *mecA*-negative while displaying phenotypic resistance, suggesting the absence of borderline oxacillin-resistant strains or alternative resistance mechanisms such as β-lactamase hyperproduction. Although *mecC* was not included in the molecular panel, the presence of undetected mecC-harboring isolates cannot be excluded and is acknowledged as a methodological limitation.

Overall, the combined application of culture-based screening, disk diffusion testing, and PCR-based detection demonstrates that molecular methods remain the most reliable approach for identifying methicillin resistance in *Staphylococcus* isolates obtained from companion animals.

## DISCUSSION

### Status of MRSA and MRSP in companion animals in Indonesia

In Indonesia, research on MRSA has largely focused on livestock-associated strains, food-chain contamination, and human hospital-acquired isolates [16, 19, 22], whereas molecular investigations involving companion animals remain scarce. Waruwu *et al*. [23] reported MRSA in cats from a veterinary clinic in Sidoarjo, East Java, where *S. aureus* was isolated from 82% of sampled cats and 8.5% were identified as MRSA. In our previous study, 4.5% (6/11) of isolates from companion animals with clinical signs of bacterial infection in Yogyakarta were classified as MRSA, while 27.2% (3/11) were identified as MRSP [24]. Overall, data regarding the occurrence of MRSA and MRSP in companion animals in Indonesia remain limited.

### Phenotypic characteristics and biochemical differentiation

Swab samples were collected from companion animals exhibiting clinical signs consistent with *S. aureus* or *S. pseudintermedius* infection. Colonies of both species were capable of fermenting mannitol, producing a yellow color change on mannitol salt agar. Gram staining revealed clusters of Gram-positive cocci. Differentiation between *S. aureus* and *S. pseudintermedius* based solely on phenotypic characteristics is challenging due to their close resemblance. However, biochemical assays such as ortho-nitrophenyl-β-galactoside (ONPG) and VP tests provide useful discriminatory value [18, 25, 26]. Both species are catalase-positive; however, *S. aureus* produces acetoin and is VP-positive, whereas *S. pseudintermedius* is VP-negative. Conversely, *S. pseudintermedius* is ONPG-positive, while *S. aureus* is ONPG-negative.

### Molecular confirmation of *S. aureus*

Phenotypically identified isolates were subsequently subjected to molecular confirmation. In this study, *S. aureus* was confirmed by PCR amplification of the *23S rRNA* and *nuc* genes. The 23S rRNA gene encodes a conserved structural component of the bacterial ribosome, making it a reliable target for species-level identification [17]. The *nuc* gene encodes thermonuclease, a virulence-associated enzyme that serves as a hallmark marker of *S. aureus* and enables differentiation from coagulase-negative staphylococci [22]. Amplification of these targets yielded the expected amplicon sizes of 1250 bp (23S rRNA) and 279 bp (*nuc*), confirming that 41 isolates were *S. aureus* (Figure 2).

### Molecular identification of *S. pseudintermedius*

Identification of *S. pseudintermedius* was performed using PCR–RFLP analysis of the *pta* gene with the *Mbo*I restriction enzyme. This method allows differentiation of *S. pseudintermedius* from other members of the *Staphylococcus intermedius* group (SIG). The *pta* gene of *S. pseudintermedius* contains an *Mbo*I restriction site that is absent in other SIG species; therefore, digestion produces two characteristic fragments of 213 bp and 107 bp, whereas other SIG members remain undigested [18, 20]. All 14 isolates displayed the expected two-band pattern, confirming their identity as *S. pseudintermedius* (Figure 3).

### Host species and anatomical distribution of isolates

In total, 55 isolates were successfully characterized, comprising 41 *S. aureus* and 14 *S. pseudintermedius* isolates derived from dogs, cats, and rabbits (Figure 4). The inclusion of rabbits as clinical cases adds novelty, as MRSA and MRSP epidemiology in small mammals remains underexplored both regionally and globally. The relatively high rate of *S. aureus* isolation from rabbits may be linked to their susceptibility to skin lesions and husbandry-related stress, which can facilitate colonization. The predominance of nasal isolates, particularly in cats, supports previous findings that the nasal cavity serves as a major reservoir and transmission source in both veterinary and household environments. These observations are consistent with earlier reports indicating that *S. aureus* commonly colonizes the skin, nasal passages, and urogenital tract of various animal species [4]. Similarly, *S. pseudintermedius* is frequently isolated from companion animals, especially dogs, and is associated with pyoderma, otitis externa, keratitis, metritis, and urinary tract infections [6–8]. The anatomical distribution of isolates provides insight into colonization dynamics and aids in distinguishing colonization from active infection. The combined phenotypic and molecular approach used in this study ensured accurate species identification, which is essential for appropriate antimicrobial therapy and epidemiological interpretation.

### Hemolytic activity and methicillin resistance detection strategies

This study represents one of the first comparative analyses of hemolytic profiles of *S. aureus* and *S. pseudintermedius* isolates from companion animals in Indonesia. Methicillin resistance was assessed using a tiered approach consisting of initial screening on oxacillin resistance screening agar base (ORSAB), phenotypic confirmation by disk diffusion testing with cefoxitin (FOX) for *S. aureus* and oxacillin (OX) for *S. pseudintermedius*, and genotypic detection of the *mecA* gene. ORSAB screening classified 95% of S. aureus isolates as MRSA and 5% as MSSA, while 79% of *S. pseudintermedius* isolates were classified as MRSP and 21% as MSSP. Disk diffusion testing identified 41% of isolates as MRSA and 43% as MRSP, whereas PCR detection of *mecA* confirmed 66%, 34%, 93%, and 7% of MRSA, MSSA, MRSP, and MSSP isolates, respectively.

### Diagnostic discrepancies and limitations of phenotypic methods

The observed variation among diagnostic methods aligns with previous reports demonstrating that ORSAB screening frequently overestimates MRSA prevalence compared with molecular confirmation. Blanc *et al*. [27] reported that only 42% of ORSAB-positive isolates were confirmed as MRSA by molecular methods. This discrepancy arises because non-*S. aureus* species can ferment mannitol and exhibit oxacillin resistance. Consequently, ORSAB should be used strictly as a preliminary screening tool and must be followed by molecular confirmation. In this study, only 66% of isolates classified as MRSA by ORSAB were *mecA*-positive, highlighting the limitations of culture-based detection and reinforcing the importance of molecular diagnostics in Indonesian veterinary laboratories.

### Silent *mecA* carriage and resistance heterogeneity

Notably, 23 *S. aureus* and *S. pseudintermedius* isolates were *mecA*-positive yet remained phenotypically susceptible to cefoxitin or oxacillin. This phenomenon suggests the presence of silent or low-expression *mecA* elements in pet-associated staphylococci, an underreported issue in Indonesia. Similar genotype–phenotype discrepancies have been described previously [28]. Silent resistance genes may remain unexpressed under routine testing conditions but pose a latent risk due to their potential for horizontal transfer. Kang *et al*. [29] described *S. pseudintermedius* isolates carrying *mecA* that were oxacillin-susceptible, referring to them as pre-MRSP strains. These isolates possess an intact mecR1–mecI regulatory system that represses *mecA* transcription. The *mecI* gene encodes a repressor protein, while mecR1 functions as a membrane-bound signal transducer; when functional, these regulators suppress *mecA* expression, resulting in phenotypic susceptibility despite gene carriage [30, 31].

### Alternative mechanisms of methicillin resistance

Conversely, eight isolates exhibited phenotypic resistance to cefoxitin or oxacillin but were *mecA*-negative. Phenotypic assays depend on bacterial growth conditions and may be influenced by agar composition, antibiotic diffusion, and inoculum density. Variations in agar depth or antibiotic potency can alter inhibition zones, leading to false resistance profiles. Heteroresistance, in which bacterial subpopulations express variable resistance levels, may also contribute to inconsistent phenotypic results. Furthermore, methicillin resistance is not exclusively mediated by *mecA*; alternative genetic determinants such as *mecC*, *fem*, and *aux* genes may also confer β-lactam resistance [32].

### AMR patterns and MDR

Antimicrobial susceptibility testing against 11 antibiotics revealed the highest resistance rates to penicillin, detected in 78% of *S. aureus* and 93% of *S. pseudintermedius* isolates. Resistance was also observed to ampicillin, amoxicillin, tetracycline, clindamycin, cefoxitin, erythromycin, oxacillin, gentamicin, vancomycin, and ciprofloxacin. These results align with global trends indicating increasing β-lactam resistance among Staphylococcus species isolated from companion animals. As noted by Ventola [33], inappropriate antimicrobial use, including underdosing, overuse, and prolonged treatment duration, creates selective pressure that promotes resistance development.

### One Health implications and study limitations

Overall, this study demonstrates a high prevalence of AMR and MDR among *S. aureus* and *S. pseudintermedius* isolated from companion animals. Isolates were classified as MDR when resistance was observed to at least one agent in three or more antimicrobial classes [26]. The comparable MDR burden observed in both species suggests convergent resistance evolution in Indonesian companion animals, a finding not previously documented in regional studies. The emergence of MRSA and MRSP in pets is of substantial concern due to their zoonotic potential and capacity to act as reservoirs of resistance genes at the animal–human–environment interface. By providing molecular evidence of MRSA and MRSP circulation in companion animals, this study strengthens the One Health perspective on AMR transmission in Indonesia.

To the best of our knowledge, this study is the first to integrate multispecies clinical sampling, molecular species confirmation, and comprehensive AMR profiling of MRSA and MRSP in Indonesian veterinary clinics. Nevertheless, several limitations should be acknowledged, including the absence of *SCCmec* or multilocus sequence typing, lack of sequencing-based confirmation, and omission of *mecC* screening, which may restrict detailed characterization of resistance mechanisms. Additionally, risk factor analysis and larger sample sizes were not included. Future studies should incorporate broader geographic sampling, longitudinal surveillance, and expanded molecular approaches to better elucidate transmission dynamics and resistance evolution.

## CONCLUSION

This study demonstrated a substantial burden of methicillin-resistant and multidrug-resistant staphylococci among companion animals with clinical infections in Indonesia. Of 55 confirmed isolates, 41 were identified as *S. aureus* and 14 as *S. pseudintermedius*, with a high prevalence of MRSA (66%) and MRSP (93%) based on *mecA* detection. MDR was widespread, affecting 93% of both *S. aureus* and *S. pseudintermedius* isolates, with particularly high resistance to β-lactam antibiotics, including penicillin, ampicillin, and amoxicillin. Notably, a considerable proportion of isolates carried *mecA* while remaining phenotypically susceptible, highlighting the presence of silent or low-expression resistance determinants that may escape routine diagnostic detection.

The findings have direct implications for veterinary clinical practice in Indonesia. Reliance solely on phenotypic methods may lead to underdiagnosis of MRSA and MRSP, potentially resulting in inappropriate antimicrobial therapy and treatment failure. The high MDR rates observed emphasize the need for evidence-based antimicrobial selection and strengthened antimicrobial stewardship in veterinary clinics. Routine incorporation of molecular diagnostics, particularly *mecA* detection, would improve diagnostic accuracy and guide more effective therapeutic decision-making. From a One Health perspective, the circulation of MRSA and MRSP in pets represents a tangible zoonotic risk for owners and veterinary personnel, underscoring the importance of infection control measures in veterinary hospitals and households.

A major strength of this study is the integrated diagnostic approach combining phenotypic identification, molecular species confirmation, and genotypic detection of methicillin resistance. The use of PCR–RFLP targeting the *pta* gene enabled accurate differentiation of *S. pseudintermedius* from other members of the Staphylococcus intermedius group, a method rarely applied in Indonesian veterinary diagnostics. Inclusion of multiple companion animal species, including rabbits, adds novelty and expands the epidemiological understanding of methicillin-resistant staphylococci beyond dogs and cats. The parallel assessment of screening, phenotypic, and genotypic methods allowed critical evaluation of diagnostic discrepancies relevant to routine laboratory practice.

Several limitations should be acknowledged. MIC testing was not performed, limiting quantitative assessment of resistance levels. Molecular characterization was restricted to *mecA* detection; *SCCmec* typing, multilocus sequence typing, WGS, and screening for *mecC* were not conducted, which constrains deeper insight into clonal relatedness and resistance evolution. The study design was cross-sectional with a relatively limited sample size and geographic coverage, and risk factor analysis was not included. These factors may limit the generalizability of the findings to the broader Indonesian companion animal population.

Future studies should incorporate larger, multicenter sampling across diverse geographic regions in Indonesia and include longitudinal surveillance to assess temporal trends in MRSA and MRSP prevalence. Expanded molecular analyses, including *SCCmec* typing, sequence-based methods, and detection of alternative resistance determinants, would enhance understanding of transmission dynamics and evolutionary pathways. Integration of risk factor analysis involving animal management, antimicrobial use history, and human–animal contact patterns would further strengthen One Health–oriented surveillance frameworks.

In conclusion, this study provides the first comprehensive molecular and phenotypic evidence of widespread MRSA and MRSP among companion animals with clinical infections in Indonesia. The high prevalence of MDR and the detection of silent *mecA* carriers highlight significant diagnostic and therapeutic challenges in veterinary practice. These findings underscore the urgent need for improved molecular diagnostics, rational antimicrobial use, and integrated One Health surveillance to mitigate the spread of methicillin-resistant staphylococci at the animal–human interface.

## DATA AVAILABILITY

All the generated data are included in the manuscript.

## AUTHORS’ CONTRIBUTIONS

GGA: Performed the experiment, data analysis, and wrote the manuscript; SIO: Conceptualization, funding, supervision, and wrote the manuscript. FBL and RW: Data analysis and manuscript review; ARPY, YRS, and MW: experiments and data analysis; All authors have read and approved the final manuscript.

## References

[1] Markey BK, Leonard FC, Archambault M (2013). Clinical Veterinary Microbiology.

[2] Cheung GYC, Bae JS, Otto M (2021). Pathogenicity and virulence of *Staphylococcus aureus*. Virulence.

[3] Khairullah AR, Sudjarwo SA, Effendi MH, Ramandinianto SC, Gelolodo MA, Widodo A, Riwu KHP, Kurniawati DA (2023). Pet animals as reservoirs for spreading methicillin-resistant *Staphylococcus aureus* to human health. J Adv Vet Anim Res.

[4] Saeed MM, Yasir JOA, Hussein AN, Hassan RM (2022). A review of animal diseases caused by staphylococci. Rev Latinoam Hipertens.

[5] Roberts E, Nuttall TJ, Gkekas G, Mellanby RJ, Fitzgerald JR, Paterson GK (2024). Not just in man's best friend:A review of *Staphylococcus pseudintermedius* host range and human zoonosis. Res Vet Sci.

[6] Sato Y, Hatayama N, Suzuki Y (2024). *Staphylococcus pseudintermedius* ST2660 isolated from a cat has strong biofilm-forming ability and increases biofilm formation at cat's normal body temperature. Sci Rep.

[7] Lynch SA, Helbig KJ (2021). The complex diseases of *Staphylococcus pseudintermedius* in canines:Where to next?. Vet Sci.

[8] Huber D, Šoštarić-Zuckermann IC, MihokovićBuhin I, Habuš J, Štritof Z, Stevanović V, Grabarević Ž (2022). Pyometra associated with *Staphylococcus pseudintermedius* in two bitches. Top Companion Anim Med.

[9] Wang Z, Guo L, Yuan C (2024). *Staphylococcus pseudintermedius* induces pyroptosis of canine corneal epithelial cells by activating the ROS–NLRP3 signalling pathway. Virulence.

[10] Grönthal T, Moodley A, Nykäsenoja J, Guardabassi L, Thomson K, Rantala M (2014). Large outbreak caused by methicillin-resistant *Staphylococcus pseudintermedius* ST71 in a Finnish veterinary teaching hospital –from outbreak control to outbreak prevention. PLoS One.

[11] Kasela M, Ossowski M, Dzikoń E, Ignatiuk K, Wlazło Ł, Malm A (2023). The epidemiology of animal-associated methicillin-resistant *Staphylococcus aureus*. Antibiotics (Basel).

[12] Van Duijkeren E, Catry B, Greko C, Moreno MA, Pomba MC, Pyörälä S, Ruzauskas M, Sanders P, Threlfall EJ, Torren-Edo J, Törneke K (2011). Review on methicillin-resistant *Staphylococcus pseudintermedius*. J Antimicrob Chemother.

[13] Afnani DA, Fatih N, Effendi MH, Tyasningsih W, Khairullah AR, Kurniawan SC, Silaen OSM, Ramandinianto SC, Widodo A, Riwu KHP (2022). Profile of multidrug resistance and methicillin-resistant *Staphylococcus aureus* (MRSA) isolated from cats in Surabaya, Indonesia. Biodiversitas.

[14] Walther B, Friedrich AW, Brunnberg L, Wieler LH, Lübke-Becker A (2006). Methicillin-resistant *Staphylococcus aureus* (MRSA) in veterinary medicine:A “new emerging pathogen”?. Berl Munch Tierarztl Wochenschr.

[15] Carroll KC, Burnham CAD, Westblade LF (2021). From canines to humans:Clinical importance of *Staphylococcus pseudintermedius*. PLoS Pathog.

[16] Fitranda M, Salasia SIO, Sianipar O, Dewananda DA, Arjana AZ, Aziz F, Wasissa M, Lestari FB, Santosa CM (2023). Methicillin-resistant *Staphylococcus aureus* isolates derived from humans and animals in Yogyakarta, Indonesia. Vet World.

[17] Aziz F, Lestari FB, Nuraida S, Purwati E, Salasia SIO (2020). Detection of Staphylococcus aureus and Staphylococcus spp. directly from fresh milk of Peranakan Etawa goats by polymerase chain reaction (PCR). J Sain Vet.

[18] Bannoehr J, Franco A, Iurescia M, Battisti A, Fitzgerald JR (2009). Molecular diagnostic identification of Staphylococcus pseudintermedius. J Clin Microbiol.

[19] Clinical and Laboratory Standards Institute (2023). Performance standards for antimicrobial susceptibility testing. CLSI supplement M100.

[20] Afshar MF, Zakaria Z, Cheng CH, Ahmad NI (2023). Prevalence and multidrug-resistant profile of methicillin-resistant *Staphylococcus aureus* and methicillin-resistant *Staphylococcus pseudintermedius* in dogs, cats, and pet owners in Malaysia. Vet World.

[21] Magiorakos AP, Srinivasan A, Carey RB, Carmeli Y, Falagas ME, Giske CG, Harbarth S, Hindler JF, Kahlmeter G, Olsson-Liljequist B, Paterson DL, Rice LB, Stelling J, Struelens MJ, Vatopoulos A, Weber JT, Monnet DL (2012). Multidrug-resistant, extensively drug-resistant and pandrug-resistant bacteria:an international expert proposal for interim standard definitions for acquired resistance. Clin Microbiol Infect.

[22] Juwita S, Indrawati A, Damajanti R, Safika S, Mayasari NLPI (2022). Genetic relationship of *Staphylococcus aureus* isolated from humans, animals, environment, and Dangke products in dairy farms of South Sulawesi Province, Indonesia. Vet World.

[23] Waruwu YKK, Khairullah AR, Effendi MH, Lukiswanto BS, Afnani DA, Kurniawan SC, Silaen OSM, Riwu KHP, Widodo A, Ramandinianto SC (2023). Detection of methicillin-resistant *Staphylococcus aureus* and multidrug resistance isolated from cats in animal clinic at Sidoarjo District, East Java, Indonesia. Biodiversitas.

[24] Alhadz GG, Salasia SIO, Yosyana ARP, Wasissa M, Lestari FB, Widayanti R (2025). Isolasi dan identifikasi methicillin-resistant *Staphylococcus aureus* dan methicillin-resistant *Staphylococcus pseudintermedius* pada kasus klinis anjing dan kucing di Yogyakarta. J Sain Vet.

[25] Bibby HL, Brown KL (2021). Identification of *Staphylococcus pseudintermedius* isolates from wound cultures by matrix-assisted laser desorption ionization–time of flight mass spectrometry improves accuracy of susceptibility reporting at an increase in cost. J Clin Microbiol.

[26] Moses IB, Santos FF, Gales AC (2023). Human colonization and infection by *Staphylococcus pseudintermedius*:An emerging and underestimated zoonotic pathogen. Microorganisms.

[27] Blanc DS, Wenger A, Bille J (2003). Evaluation of a novel medium for screening specimens from hospitalized patients to detect methicillin-resistant *Staphylococcus aureus*. J Clin Microbiol.

[28] Stasiak M, Maćkiw E, Kowalska J, Kucharek K, Postupolski J (2021). Silent genes:Antimicrobial resistance and antibiotic production. Pol J Microbiol.

[29] Kang MH, Chae MJ, Yoon JW, Kim SG, Lee SY, Yoo JH, Park HM (2014). Antibiotic resistance and molecular characterization of ophthalmic Staphylococcus pseudintermedius isolates from dogs. J Vet Sci.

[30] Berger-Bächi B, Rohrer S (2002). Factors influencing methicillin resistance in staphylococci. Arch Microbiol.

[31] Petinaki E, Arvaniti A, Dimitracopoulos G, Spiliopoulou I (2001). Detection of *mecA*, *mecR1*, and *mecI* genes among clinical isolates of methicillin-resistant staphylococci by combined polymerase chain reactions. J Antimicrob Chemother.

[32] Fishovitz J, Hermoso JA, Chang M, Mobashery S (2014). Penicillin-binding protein 2a of methicillin-resistant *Staphylococcus aureus*. IUBMB Life.

[33] Ventola CL (2015). The antibiotic resistance crisis:part 1:causes and threats. P T.

